# In-depth analysis of the expression and functions of signal transducers and activators of transcription in human ovarian cancer

**DOI:** 10.3389/fonc.2022.1054647

**Published:** 2022-11-29

**Authors:** Xiaodi Gong, Xiaojun Liu

**Affiliations:** Department of Gynaecology and Obstetrics, Changzheng Hospital, Naval Medical University, Shanghai, China

**Keywords:** bioinformatics, LASSO, STAT5A, cell invasion, ovarian cancer

## Abstract

**Background:**

Signal transducers and activators of transcription (STAT) transcription factors, a family of genes encoding transcription factors, have been linked to the development of numerous types of tumors. However, there is a relative paucity of a comprehensive investigation of the expression and functional analysis of STATs in ovarian cancer (OV).

**Method:**

Gene expression profile interaction analysis (GEPI2A), Metascape, The Cancer Genome Atlas (TCGA), Kaplan-Meier Plotter, Linkedomics, and CancerSEA databases were used for expression analysis and functional enrichment of STATs in ovarian cancer patients. We screened potential predictive genes and evaluated their prognostic value by constructing the minor absolute shrinkage and selection operator (LASSO) Cox proportional risk regression model. We explored STAT5A expression and its effects on cell invasion using ovarian cancer cells and a tissue microarray.

**Results:**

The expression level of STAT1 was higher, but that of STAT2-6 was lower in cancerous ovarian tissues compared to normal tissues, which were closely associated with the clinicopathological features. Low STAT1, high STAT4, and 6 mRNA levels indicated high overall survival. STAT1, 3, 4, and 5A were collectively constructed as prognostic risk models. STAT3, and 5A, up-regulating in the high-risk group, were regarded as risk genes. In subsequent validation, OV patients with a low level of P-STAT5A but not low STAT5A had a longer survival time (*P*=0.0042). Besides, a negative correlation was found between the expression of STAT5A and invasion of ovarian cancer cells (R= -0.38, *p* < 0.01), as well as DNA repair function (R= -0.36, *p* < 0.01). Furthermore, transient overexpression of STAT5A inhibited wound healing (21.8%, *P*<0.0001) and cell migration to the lower chamber of the Transwell system (29.3%, *P*<0.0001), which may be achieved by regulating the expression of MMP2.

**Conclusion:**

It is suggested that STAT1, STAT4, and STAT6 may be potential targets for the proper treatment of ovarian cancer. STAT5A and P-STAT5A, biomarkers identified in ovarian cancer, may offer new perspectives for predicting prognosis and assessing therapeutic effects.

## Introduction

Among gynecological tumors, ovarian cancer is the leading cause of death. About 19,880 new cases of ovarian cancer will be diagnosed in the United States in 2022, the equivalent of about 54 new cases each day, and 12,810 deaths from ovarian cancer are projected to occur, approximately 35 deaths per day (1). Because ovarian cancer can be divided into at least five histological subtypes, accompanied by unique risk factors, origin cells, and genomic characteristics, it cannot be detected early in population-based screening and is usually diagnosed late (2). Upfront treatment mainly depends on cytoreductive surgery without residual disease and platinum-based chemotherapy, and anti-angiogenic agents are added in patients with stage IV and recurrence (3). However, recurrent cancer is often resistant to platinum chemotherapy, which leads to a lack of effective treatment. Fortunately, adding poly (ADP- ribose) polymerase (PARP) molecular inhibitors to recurrent patients with BRCA1/BRCA2 mutations has made significant progress in maintenance therapy (4). The combined treatment of multiple methods can slowly increase the 5-year survival rate of ovarian cancer, but the prognosis is still not significantly improved.

STAT transcription factors (STATs) were discovered in 1994 (5). Seven STATs family members are found in mammals with similar structural and functional characteristics, all encoded by their genes: STAT1 (chromosome position: 2q32.2), STAT2 (12q13.3), STAT3 (17q21.2), STAT4 (2q32.2), STAT5A (17q21.2), STAT5B (17q21.2) and STAT6 (12q13.3) (6). Each of them played unique roles in signal transduction. The Janus kinase (JAK) and STAT pathways are involved in the biological effects of more than 50 cytokines and growth factors (7). Activated JAK phosphorylates the conserved c-terminal tyrosine residue in STATs, facilitating them to form dimerization, which leads to the activation of STATs and then translocation into the nucleus through Ran-GTP-dependent mechanisms. Subsequently, STATs bind to specific target DNA promoter sequences to control corresponding gene transcription (5). In this way, the translocation of STATs from the cytoplasm to the nucleus realizes the transmission of extracellular signals. It then affects the expression of target genes to regulate cell proliferation, differentiation, apoptosis, and angiogenesis (8).

The activation of STATs in normal signal transduction is rapid and transient, and the sustained activation of STATs is closely related to the process of malignant transformation. Tumors of various types exhibit abnormal activation of STAT family members, including ovarian cancer (9), breast cancer (10), prostate cancer, and (11) hematological and head and neck cancer (12), of which have been confirmed to be involved in angiogenesis, invasion, and metastasis of tumor cells, as well as their escape from the immune system.

STAT1 played a dual role in ovarian cancer. For instance, a positive effect of STAT1 in ovarian cancer was that it upregulated the expression of inducible nitric oxide synthase (iNOS) (13), resulting in the release of cytotoxic nitric oxide (NO) (14) and accelerating the progression of the disease (15); however, NO could also promote ovarian cell apoptosis by increasing the expression of p53 (16). The contradictory role of STAT1 in promoting and inhibiting cancer also existed in invasion and metastasis (17, 18), angiogenesis (19), immunologic responsiveness (20), and chemotherapeutic drug reactivity of ovarian cancer (21).

The Fibrillin-1/VEGFR2/STAT2 signal axis modulated the process of glycolysis and angiogenesis by activating STAT2, which induced cisplatin resistance in ovarian cancer cells (22). Activated STAT3 facilitated migration and invasion of ovarian cancer by inducing the expression of MMP2 and MMP9 (23, 24), and assisting in the epithelial-to-mesenchymal transition (EMT) process of ovarian cancer (25). STAT3 regulated the expression of HIF-1α (26), contributing to ovarian cancer angiogenesis. In addition, ovarian cancer cells expressing STAT3 showed increased resistance to chemotherapy (27) and with cancer stem cells (CSCs) or CSC-like phenotypes (28). Likewise, STAT4 could induce activation of tumor-associated fibroblasts (CAF) through tumor-derived Wnt7a, which promoted peritoneal metastasis of ovarian cancer through the EMT process (29). Overexpression of human epidermal growth factor receptor 4 (HER4) in ovarian CSCs mediated STAT5 activation to enhance the survival and growth of ovarian CSCs (30). Upon oncoproteomic analysis, STAT5B was overexpressed in ovarian cancer that recurred after chemotherapy. Further research confirmed that STAT5B and RELA (NF-kappaB p65) were responsible for carboplatin resistance in ovarian carcinoma (31). Moreover, the decreased STAT5B led to CD8^+^ effector memory T (T_EM_) cell dysfunction in ascites of high-grade serous ovarian cancer patients, thus causing shortened relapse-free survival (RFS) (32). Collagen triple helix repeat containing 1(CTHRC1), secreted by epithelial ovarian Cancer (EOC) cells, promoted M2-like polarization of tumor-associated macrophages (TAMs) by activating STAT6. As a result, this facilitated EOC cell invasion and migration (33). Additionally, STAT6 was also involved in the stemness maintenance and function of ovarian CSCs (34).

**SCHEME 1:**
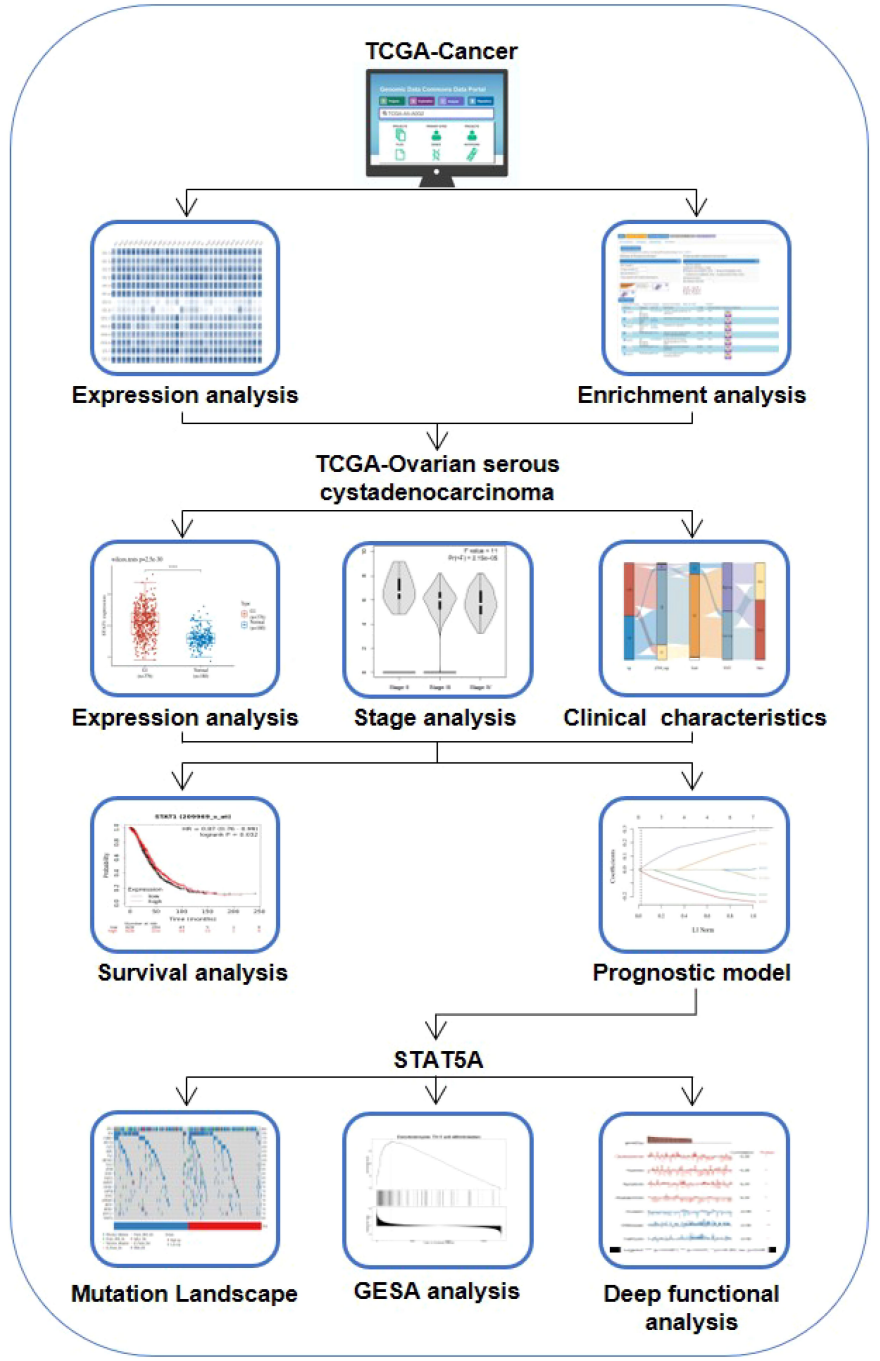
Protocol for investigating the role of STATs in ovarian cancer.

Although there are partial reports on the role of individual STAT in the development and progression of ovarian cancer, the role of the entire STATs family in ovarian cancer has not been explored through bioinformatics. Here, a detailed analysis of STAT transcription factor expression in ovarian cancer was performed, and potential biomarkers were identified. We sought to ascertain the pattern of expression, potential biological function, and unique prognostic significance of STATs in ovarian cancer (**Scheme 1**).

## Results

### The main functions of the STAT family

**Figure 1:**
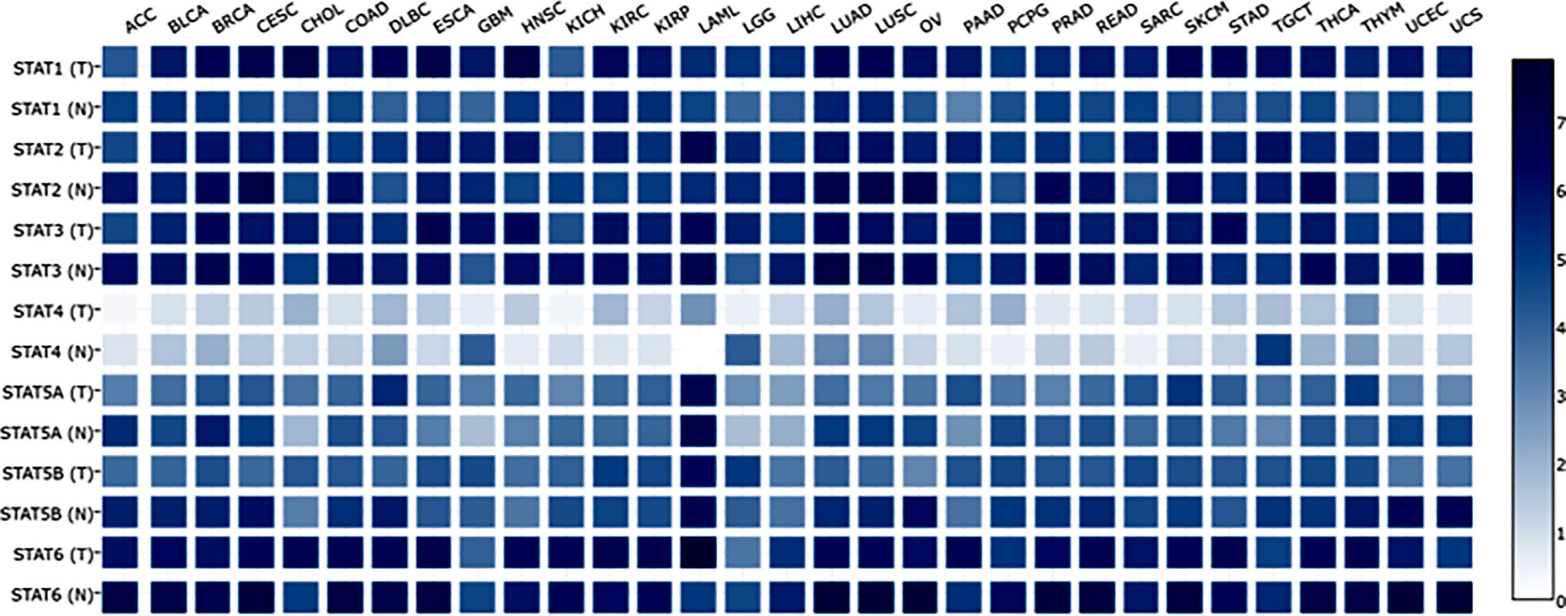
Expression matrix plots of STAT family members in various cancers. Abbreviations for tumor names are annotated above the plot. Their specific tumor names have been annotated one by one as follows. STAT family members from tumor tissues (T) and the normal counterpart (N) were enumerated on the left. The color bar at right is presented in log_2_-scale and began at zero, which is indicated the expression level of the STATs genes. ACC: Adrenocortical carcinoma; BLCA: Bladder Urothelial Carcinoma; BRCA: Breast invasive carcinoma; CESC: Cervical squamous cell carcinoma and endocervical adenocarcinoma; CHOL: Cholangio carcinoma; COAD: Colon adenocarcinoma; DLBC: Lymphoid Neoplasm Diffuse Large B-cell Lymphoma; ESCA: Esophageal carcinoma; GBM: Glioblastoma multiforme; HNSC: Head and Neck squamous cell carcinoma; KICH: Kidney Chromophobe; KIRC: Kidney renal clear cell carcinoma; KIRP: Kidney renal papillary cell carcinoma: LAML: Acute Myeloid Leukemia; LGG: Brain Lower Grade Glioma; LIHC: Liver hepatocellular carcinoma; LUAD: Lung adenocarcinoma; LUSC: Lung squamous cell carcinoma; MESO: Mesothelioma; OV: Ovarian serous cystadenocarcinoma; PAAD: Pancreatic adenocarcinoma; PCPG: Pheochromocytoma and Paraganglioma; PRAD: Prostate adenocarcinoma; READ: Rectum adenocarcinoma; SARC: Sarcoma; SKCM: Skin Cutaneous Melanoma; STAD: Stomach adenocarcinoma; TGCT: Testicular Germ Cell Tumors; THCA: Thyroid carcinoma; THYM: Thymoma; UCEC: Uterine Corpus Endometrial Carcinoma; UCS: Uterine Carcinosarcoma; UVM: Uveal Melanoma.

**Figure 2:**
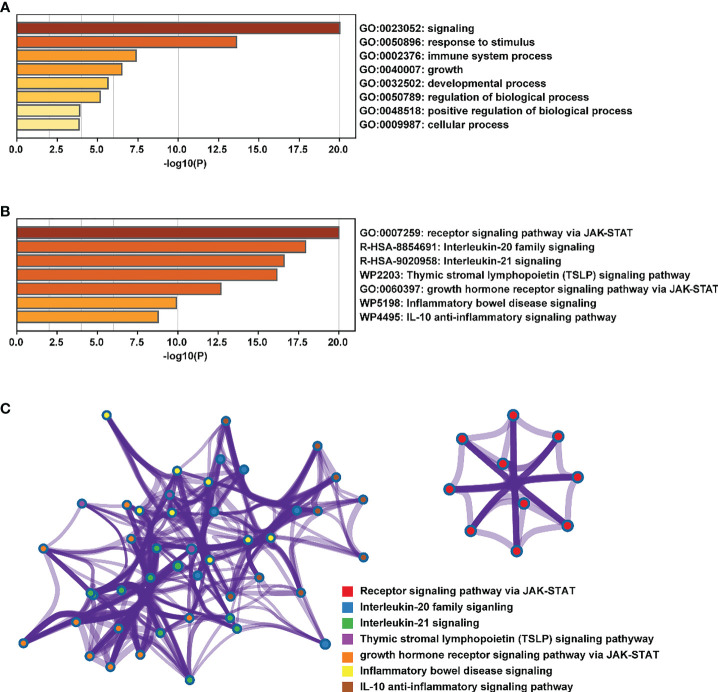
Enrichment analysis of the main functions of the STAT family. **(A, B)** biological processes and pathways related to STATs genes as enriched in Gene Ontology, colored by *p*-values. **(C)** An enrichment network: each node in a cluster is colored accordingly, with cluster IDs that are close together being grouped.

At present, researchers have identified seven STAT transcription factors in mammalian cells. A comparison was made between STAT transcription in cancers and normal tissues based on the gene expression profiling interactive analysis (GEPIA2) database (http://gepia2.cancer-pku.cn/#analysis). Selecting “TCGA normal+ GTEx normal” as the matched normal tissue data, **Figure 1** shows the expression of STAT family members in 31 different tumors (T) and paired normal tissue (N), plotted using log2(TPM + 1) transformed expression data. Subsequently, the Metascape database was used for enrichment analysis of significant functions of 7 STAT family genes including STAT1, STAT2, STAT3, STAT4, STAT5A, STAT5B, and STAT6 (http://metascape.org/gp/index.html#/main/step1). Enrichment standards were as follows: an enrichment factor of >1.5, a minimum count of 3, and a *p*-value of 0.01. We found that the STAT transcription factors family played crucial roles in biological processes such as signaling, response to stimulus, immune system process, growth, developmental process, regulation of biological processes, positive regulation of biological processes, and cellular processes **(** **Figure 2A** **)**. Moreover, **Figure 2B** **and** **Supplementary Material Table 1** showed the top-level significantly enriched signal pathways, including receptor signaling pathway *via* JAK-STAT, Interleukin-20 family, Interleukin-21 signaling, Thymic stromal lymphopoietin (TSLP) signaling pathway growth hormone receptor signaling pathway *via* JAK-STAT, inflammatory bowel disease signaling, and IL-10 anti-inflammatory signaling pathway. The above-enriched signal pathways were shown in the form of a network in **Figure 2C** to understand the relationship between these GO terms. Edges were formed between terms with a similarity > 0.3. Each node represents an enriched term and is colored by its cluster ID, where nodes sharing the same cluster-ID are usually close to each other. For clarity, only one term tag was displayed per cluster in the lower right corner, and all node tags can be checked by visualizing the network using Cytoscape or a browser. Therefore, the ligand-dependent activated STAT transcription factors family acted as a signaling hub *via* modulating downstream target genes’ expression and participating in the tumor occurrence and development.

### STAT transcription in ovarian cancer patients

**Figure 3:**
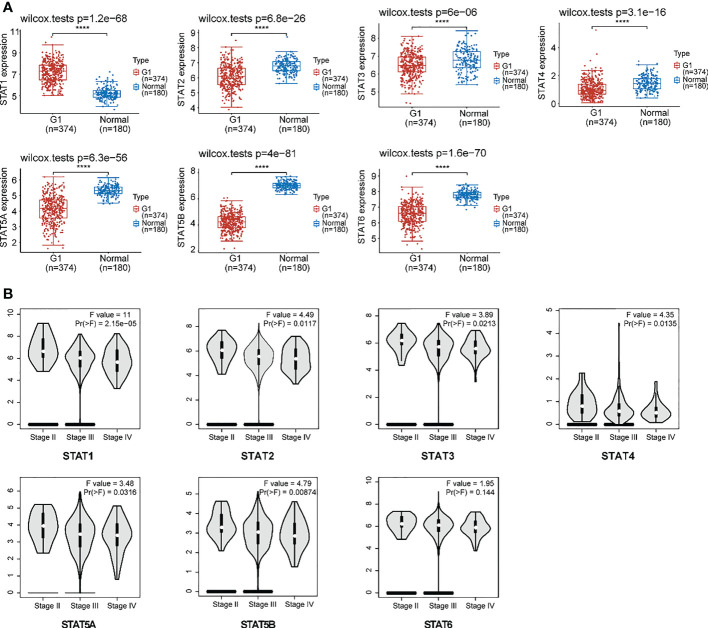
Expression of STAT family members in ovarian cancer. **(A)** The expression distribution of STAT family genes in ovarian cancer tissues (G) and normal tissues. Top-left represented the significance *P*-value, ****P < 0.0001. **(B)** An association between STATs expression and major tumor stages in patients with ovarian cancer.

Our analysis included 374 OV patients and 32 normal tissues filtered from the available data; an overview of their baseline data is provided in **Supplementary Material Table 2**. First, we assessed the expression of the STAT transcription factor by comparing ovarian cancer with normal ovarian tissues. According to research, ovarian cancer tissues exhibited higher STAT1 but lowered STAT2-6 expression than normal tissues (**Figure 3A**). Moreover, there was a positive correlation between the gene expression of different STAT family members in OV (**Figure S1**). Using the GEPIA2 database (http://gepia2.cancer-pku.cn/#analysis), an analysis was also performed of the association between the expression of STATs in ovarian cancer and major tumor stages. The results indicated that, in contrast to STAT6, the expression of other STATs family members varied significantly (**Figure 3B**). Based on the above results, STAT members exhibited different expression patterns in ovarian cancer and seemed involved in various phases of ovarian development.

### Expression distribution trend of STATs for different clinical characteristics of ovarian cancer patients

**Figure 4:**
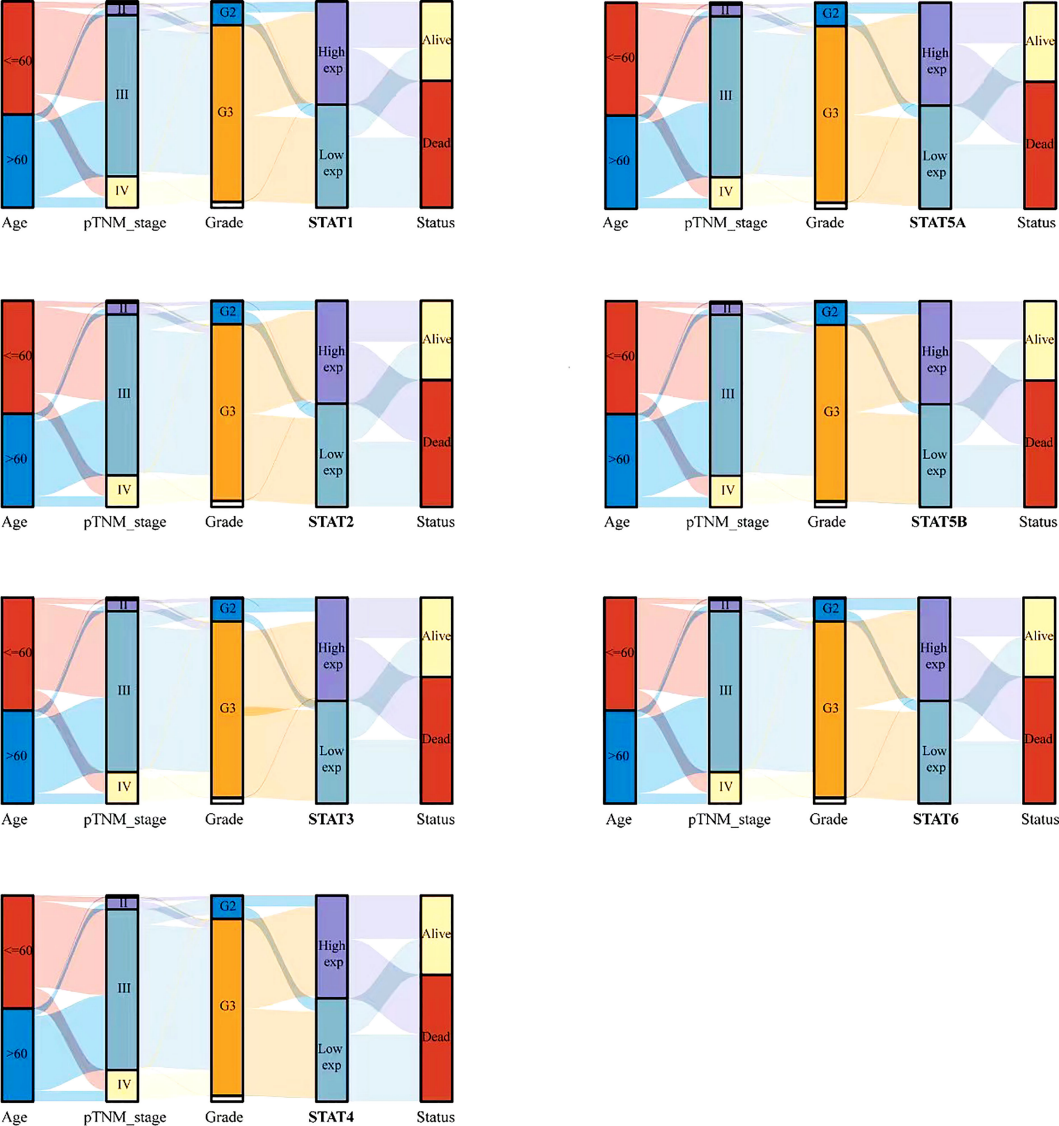
Relationship between STAT family and clinical characteristics of ovarian cancer patients. Rows represent feature variables, different color represents different age (<=60 years, >60 years) or pTNM_stage (I, II, III, IV) or Grade (G1, G2, G3) or expression level (High exp, Low exp) or survival status (Alive, Dead). Lines show how the same sample is distributed across different feature variables.

To further study the relationship between STAT family and tumor stage and grade, a Sankey diagram was drawn (**Figure 4**), which showed the distribution trend between different clinical characteristics, including age, tumor stage, grade, and the expression of STAT gene family member, and the survival status of ovarian cancer patients. There were five columns representing age, pTNM_stage, Grade, STAT1-6 expression, and survival Status in each figure, respectively. Different colors represented different ages (<= 60 years and > 60 years), pTNM_stages (I, II, III, IV), Grades (G1, G2, G3), expression levels of STAT1-6 (High exp, Low exp), Status (Alive, Dead). The above variables are connected by connecting lines to obtain the distribution of the same ovarian cancer sample across various characteristics. Through the plotting of these diagrams, we can see that patients with advanced (III, IV) ovarian cancer were more likely to have low expression of STAT family members. Differentially, in high-grade (G3, G4) ovarian cancer, STAT1, 2, 4, and 5A were highly expressed, while STAT3 and STAT5B were mostly lowly expressed. In addition, the low expression group of other STAT members except STAT5B had more deaths. This reflected the complexity of the role of different STAT members in the occurrence and development of ovarian cancer.

### Association of the expression of STATs with the prognosis of ovarian cancer patients

**Figure 5:**
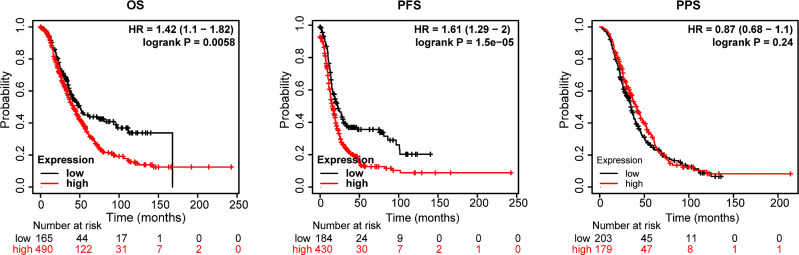
Relationship between the STATs family and survival of ovarian cancer patients. Probe Id (Gene symbol): 200887_s_at (ISGF-3, STAT91, STAT1), 225636_at (STAT2), 225289_at (STAT3), 206118_at (STAT4), 203010_at (STAT5, MGF, STAT5A), 212549_at (STAT5B), 201331_s_at (STAT6, IL-4-STAT, D12S1644). OS, overall survival; PFS, progression-free survival; PPS, post-progression survival, HR= hazard ratio.

Next, an assessment was made of the influence of STATs on ovarian cancer survival. According to openly accessible data (2021 version: http://kmplot.com/analysis/index. Php?p=service&cancer=ovar), using Kaplan-Meier Plotting tools, we investigated whether mRNA levels of STATs correlated with the survival time of ovarian cancer patients by “mean expression of selected genes” in multiple genes option. The desired Affy ID is valid: 200887_s_at (ISGF-3, STAT91, STAT1), 225636_at (STAT2), 225289_at (STAT3), 206118_at (STAT4), 203010_at (STAT5, MGF, STAT5A), 212549_at (STAT5B), 201331_s_at (STAT6, IL-4-STAT, D12S1644). Compared with the high-expression group of the STATs family, the overall survival (OS) and progression-free survival (PFS) of ovarian cancer patients in the low-expression group were higher in **Figure 5**. The median survival time (MST) for OS and PFS was 41.87 months *versus* 50.03 months (HR=1.42, *P*=0.0058), and 16 months *versus* 22.24 months (HR=1.61, *P*=1.5×10^-5^) in the high expression/low expression cohort, respectively. While the effect on post-progression survival (PPS) was not significant (HR=0.87, *P*=0.24, MST: 41 months *versus* 35 months). The whole high expression level of STATs transcription factors increased the risk of ovarian cancer death by 1.42 times. Therefore, the mean low expression of STATs members is beneficial to the survival of ovarian cancer patients.

Besides, the effect of each STAT member on the survival time of ovarian cancer patients was also analyzed using the same Probe Id as above (**Table 1**). Based on Kaplan-Meier curves and log-rank tests, the results in **Figure 5** showed that a significant correlation was observed between increased STAT4 and 6 mRNA levels, decreased STAT1 mRNA levels, and overall survival (OS) in ovarian cancer patients. (*P* < 0.05). Ovarian cancer patients with a high level of STAT4 and 6 gene expression or a low level of STAT1 gene expression had high OS.

**Table 1:** Correlation between the expression of STATs and OS or PFS in ovarian cancer patients.

	OS			PFS			
	MST (months)		HR (95% CI)	*P*-Value	MST (months)		HR (95% CI)	*P*-Value	
	Low	High			Low	High
**STAT1**	44.13	50	0.84 (0.72-0.98)	0.023	22.13	19.09	1.19 (1.04-1.37)	0.011
**STAT2**	48	40.4	1.32 (1.05-1.66)	0.016	22.6	15	1.63 (1.30-2.05)	1.7e-05
**STAT3**	40	48	0.89 (0.71-1.12)	0.32	18	16.03	1.27 (1.05-1.54)	0.016
**STAT4**	43	46	0.84 (0.73-0.96)	0.09	18.79	26.06	0.85 (0.74-0.97)	0.02
**STAT5A**	42.17	46.82	0.89 (0.77-1.02)	0.082	19.23	20.43	1.07 (0.94-1.21)	0.33
**STAT5B**	44.3	45.97	0.88 (0.78-1.01)	0.059	19.09	20.93	0.93 (0.82-1.06)	0.3
**STAT6**	43	50.3	0.79 (0.69-0.91)	0.0012	20	20	1.09 (0.96-1.25)	0.17

MST, median survival time; Low, Low expression cohort; High, High expression cohort; HR, hazard ratio, 95% CI, 95% Confidence interval.

Moreover, in ovarian cancer patients with different pathological types, STAT expression was tested for potential correlation with OS, progression-free survival (PFS) as well as post-progression survival (PPS), respectively (**Supplementary Material Tables 3**–**5** ). Patients with serous ovarian cancer expressed lower levels of STAT1 mRNA, while higher levels of STAT 2, 5A, and 5B mRNA had longer PFS but had no effect on patients with endometrioid carcinoma. Based on these above results, most members of the STAT family, except STAT3, may be promising prognostic indicators for ovarian cancer.

### Developing and evaluating a STATs prognosis prediction model

**Figure 6:**
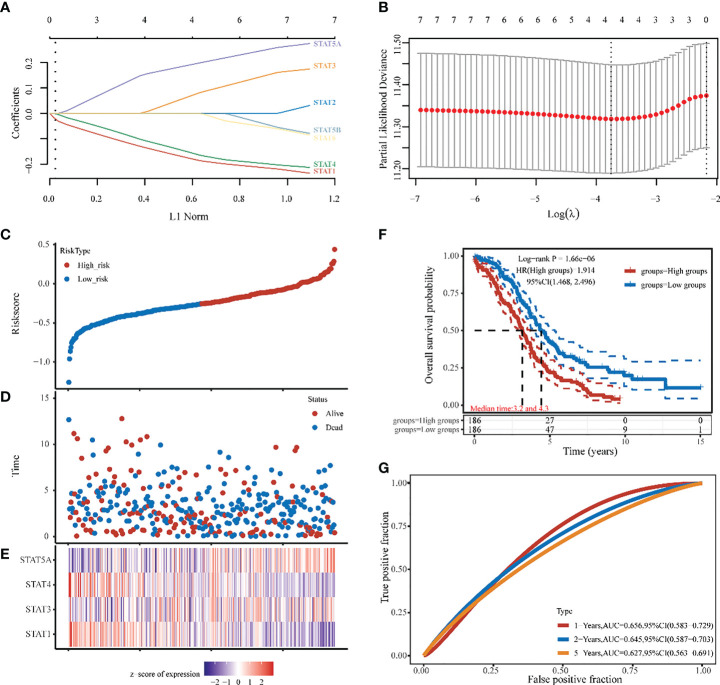
Ovarian cancer survival prediction and STAT genes selection. **(A)** The coefficients of 7 STATs in the LASSO model were screened by 10-fold cross-validation. **(B)** Analysis of the seven selected STATs by X-tile. **(C, D)** The selected dataset’s risk score, survival time, and survival status. **(E)** The heatmap was the gene expression from the signature. **(F)** A risk model for signature OV patients with Kaplan-Meier survival analysis. HR (High exp) represents the hazard ratio of the low-expression sample relatives to the high-expression sample. **(G)** ROC curves of 1,2,5 years, 1-, 2-, and 5-year overall survival probability based on the STATs Risk score.

Four STAT members with potential prognostic significance were identified by LASSO (lambda.min=0.0234). A stepwise multivariate Cox regression model was constructed using STAT1, STAT3, STAT4, and STAT5A as filter variables (**Figures 6A, B** **)**. The Risk score was calculated as follows: (-0.1694) * *STAT1* + (0.0554) * *STAT3* + (-0.1447) * *STAT4* + (0.1837) * *STAT5A*. Smooth curve fitting provided the following results, which showed the Risk score from low (blue spot) to high (blue spot), thus based on the Risk score median, a cut-off value was determined (high risk: score > -0.257, low risk: score < -0.257) (**Figure 6C** **)**. As shown in scatter plots and also Kaplan-Meier plots **Figures 6D, F** **)**, patients with a high-Risk score had a short median survival time (median time=3.2 vs. 4.3 years, hazard ratio [HR] =1.914, *P* = 1.66e-06). The heatmap was the gene expression of STAT1, 3, 4, and 5A from the signature. In the high-risk group, the protective STAT1 and STAT4 genes were low expressed, whereas STAT3 and 5A, the risk genes, were significantly higher expressed **(** **Figure 6E** **)**. In terms of time-dependent ROC curves, 1-, 2-, and 5- years of survival were assessed using Area Under Curve (AUC) values of 0.659, 0.645, and 0.627, respectively **(** **Figure 6G** **)**. A prognostic model based on disease-specific survival (DSS) was also constructed through STAT1, 4, 5A. Compared to the low-risk group with STAT1,4, the high-risk group with STAT5A was closely linked with a worse 1-, 2-, 5- years DSS of ovarian cancer patients (median time=3.4 vs. 4.7 years, HR =1.831, *P* = 3.31e-05 in **Figure S2**). Finally, as revealed by univariate analysis, Age, Race, and STAT 1, 4, and 5A were significantly related to OS based on the TCGA cohort **(** **Figure S3A** **)**. Using the factors aforementioned above, we performed a multivariate Cox regression analysis. As a result, STAT5A was still an independent predictor of outcome for this cohort of patients (hazard ratio [HR] = 1.3, *P* < 0.001), which was consistent with LASSO analysis (**Figure S3B** **)**. Additionally, **Figures 3C, D** displayed the cohort’s 1-, 2-, and 5-years OS Nomograms. As STAT5A was the gene with the highest risk score in the OV prognostic model, it had become the focus of follow-up research.

### STAT5A gene mutation analysis in ovarian cancer

**Figure 7:**
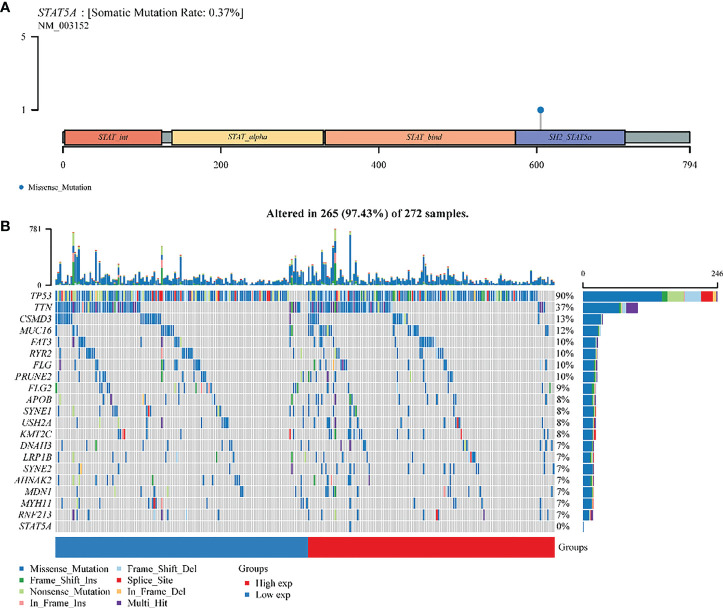
A landscape analysis of STAT5A gene mutations in ovarian cancer. **(A)** Lollipop charts of the mutated STAT5A gene. **(B)** Oncoplot displays the somatic landscape of OV TCGA cohorts. The genes and samples are sorted according to their mutation frequency and histology; Above the legend, the bar plot shows the number of mutations burdened.

STAT5A was altered (73%) in 272 samples from 374 patients with ovarian serous cystadenocarcinoma. The somatic mutation rate of STAT5A was only 0.37%, which was manifested as a gene missense mutation, leading to abnormal amino acid coding in the SH2 domain (**Figure 7A**). The panoramic waterfall mutation type diagram shows that each sample’s mutation load was different, and the median value was 82. TP53 had the highest mutation rate (90%), and the top ten mutated genes included TTN (37%), MUC16(12%), CSMD (13%), FAT3 (10%), FLG (10%), RYR2 (10%), PRUNE2 (10%). FLG2 (9%) and APOB (8%). However, STAT5A mutation only occurred in the group with high STAT5A expression, so there should be no mutation in ovarian cancer with relatively low STAT5A expression (**Figures 3**, **7B** **S4B**). A missense mutation was the main classification of gene mutation in each sample. Single nucleotide polymorphisms (SNPs) were the most common mutation type. Cytosine (C > T, C > A, C > G) and thymine (T > A, T > C, T > G) are the main types of single nucleotide mutation (SNV) mutations **(** **Figure S4A**).

### Correlation between STAT5A and the functional states of OV cells

**Figure 8:**
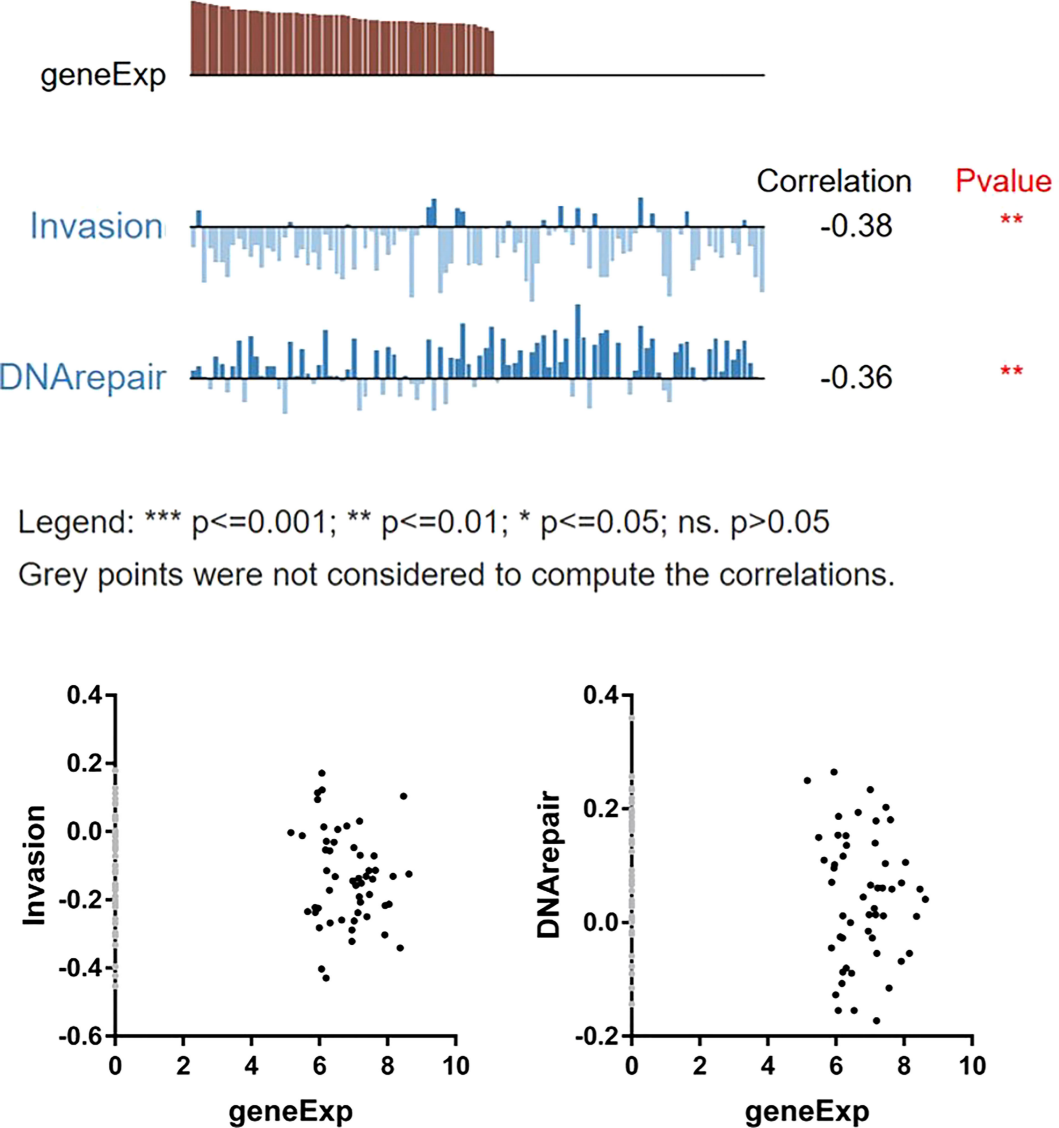
Correlation between STAT5A and functional states of OV cells.

To further study the role of STAT5A in OV, GSEA online database-Linkedomics (http://linkedomics.org) was used to explore the pathways and functions involved in STAT5A. We first analyzed the 50 most positively and negatively affecting genes related to STAT5A expression, as shown in the heat map in **Figures S5A, B**. Then GO and KEGG analysis of STAT5A in patients with OV was carried out in **Figures S5C, D** which revealed significant enrichment in mitochondrial gene expression, mitochondrial respiratory complex assembly, adaptive immune response, Oxidative phosphorylation, Chemokine signaling pathway, NF-kappa B signaling pathway, and JAK-STAT signaling pathway. From this, it can be concluded that the transcription factor STAT5A may affect the oxidative phosphorylation process of cells through the negative regulation of the mitochondrial respiratory chain complex and then interfere with the immune regulation and signal molecule transmission process of the body. Next, we conducted a more in-depth analysis of the function of STAT5A in OV using the CancerSEA single cell sequencing database (http://biocc.hrbmu.edu.cn/CancerSEA). Single gene analysis of STAT5A from different cell groups, which denoted different OV patients-derived xenograft samples, was performed. There are 7 functional states including Quiescence (R=0.28), Hypoxia (R=0.28), Apoptosis (R=0.24), Angiogenesis (R=0.23), Cell Cycle (R=-0.30), DNA repair (R=-0.36) and Invasion (R=-0.38) that are significantly related to STAT5A (*P* < 0.05, **Figure S6**). Specifically, a significant inverse relationship was found between STAT5A expression and invasive behaviors and DNA damage repair (**Figure 8**, *P* < 0.01), indicating that lower STAT5A expression could promote ovarian cancer cell invasion as well as improve the ability of cells to repair DNA damage, thus participating in the process of metastasis and recurrence of ovarian cancer.

### Analysis of the expression of STAT5A on OV tissues and cell lines

**Figure 9:**
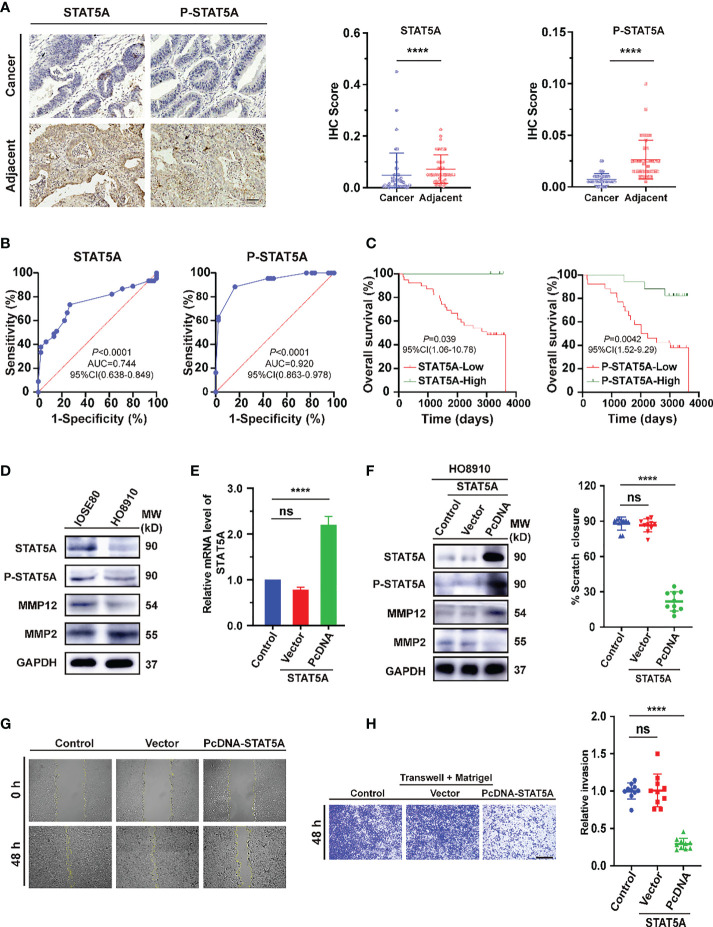
Evaluation of STAT5A expression in ovarian cancer tissues and cell lines. **(A)** Immunohistochemical staining of STAT5A and p-STAT5A on ovarian cancer and adjacent tissues from TMA samples (n=90). Black scale bar, 50 μm. **(B)** The ROC curves of STAT5A and P-STAT5A are based on independent tests. **(C)** Kaplan–Meier OS curves based on STAT5A or P-STAT5A level. **(D)** Immunoblots illustrating the basal expression of STAT5A, P-STAT5A (Tyr 694), MMP12, and MMP2 in the indicated human ovarian serous adenocarcinoma cell (HO8910) and normal ovarian epithelial cell line (IOSE80). **(E, F)** The mRNA and corresponding protein expression levels of SATAT5A were determined 48 h post-transfection with pcDNA-STAT5A plasmids in HO8910 cells. For normalization, GAPDH was used as an internal reference. MW: molecular weight. **(G)** Scratch-wound assay to quantify HO8910 cell migration ability. The scratches were recorded at 0 h and 48 h after scratching. Yellow lines indicated the scratched edges. White scale bar, 100 μm. **(H)** Transwell invasion assay with Matrigel. Cell invasion abilities were measured 48 h after cells or STAT5A overexpressing cells seeding onto a Matrigel-coated transwell filter. Black scale bar, 200 μm. **** *P*<0.0001, ns: not significantly determined by one-way ANOVA **(E, F, H)**, Mann-Whitney test **(B)**, Chi-square test, Kaplan-Meier survival analysis, and Log-rank statistical test **(C)**.

For further validation of the main conclusion in **Figure 8**, we first verified the expression of STAT5A and P-STAT5A in ovarian cancer from tissue microarray (TMA). In the detection of 45 pairs of ovarian cancer and adjacent normal tissues, the expression levels of both in cancer were significantly lower than those in para-cancerous tissues (**Figure 9A**, *P*<0.0001). The receiver operating characteristics (ROC) curves on independent tests of STAT5A and P-STAT5A are illustrated in **Figure 9B**. The optimal cut-off value for STAT5A was < 0.04375 (sensitivity 73.33%, specificity 73.33%, AUC=0.744, *P*<0.0001), while that for P-STAT5A was < 0.0125 (sensitivity 83.37%, specificity 83.72%, AUC =0.920, *P*<0.0001). Kaplan-Meier survival plots revealed that OV patients with high STAT5A expression had longer survival times than those with low STAT5A levels (*P* =0.039). However, high expression of P-STAT5A seems to be a better prognostic indicator of ovarian Cancer (*P* =0.0042, **Figure 9C**). Consistently, the univariate and multivariate Cox regression analyses of OS in paired ovarian cancer and para-cancerous tissues showed that P-STAT5A rather than STAT5A could be an independent risk factor (*P*=0.032, **Supplementary Material Table 6** in Supporting Information). Next, to address the role of STAT5A in OV cell invasiveness, human ovarian serous cell line HO8910 was used as the research object and normal ovarian epithelial cell IOSE80 as the control. First, we explored the baseline expression of transcription factor STAT5A, the activated form P-STAT5A and matrix metalloproteinases 2 (MMP2) and MMP12. The latter is involved in the degradation of extracellular matrix (ECM), in turn, mediates the epithelial-to-mesenchymal transition (EMT) process, which is known as one of the primary mechanisms for tumor invasion and metastasis. Compared to IOSE80 cells, the levels of STAT5A, P-STAT5A, and MMP12 proteins decreased significantly in HO8910 cells, while MMP2 levels increased (**Figure 9D**). After that, STAT5A overexpression plasmids were transiently transfected into HO8910 cells. The transfection efficiency was verified by quantitative real-time PCR (qRT–PCR) (**Figure 9E**, *P*<0.0001) and Western blotting analysis (**Figure 9F**). HO8910 cells overexpressing STAT5A exhibited increased MMP12 expression, while MMP2 was significantly suppressed. Compared with the control and the negative vector transfection group, a significant reduction was observed in the migration ability of cells transfected with cDNA-STAT5A. It can be seen by the area of wound-healing (marked by the yellow line in the figures) significantly decreased from the initial scratch time (0 h) to 48 h post-scratching (**Figure 9G**, *P*<0.0001).

Furthermore, HO8910 cells were seeded in the upper compartments of Matrigel-coated transwell chambers to assess cell invasion ability. After 48 h, the number of cells that invaded the lower chamber in the STAT5A-PcDNA transfected group was less than 30% of the control group (**Figure 9H**, *P*<0.0001). These results suggested that low expressed STAT5A may directly or indirectly regulate the expression of MMP2 and promote the invasion and metastasis of ovarian serous cystadenocarcinoma cells.

## Discussion

STAT5 consists of two isoforms, STAT5A and STAT5B, each encoded by a different gene, although they share 94% of the same structure (35). STAT5A was cloned from the lactation tissue of sheep in 1994 and was initially called mammary gland factor (MGF) (36), which could initiate milk protein expression and modulate prolactin action (37). As part of the classical JAK2-STAT5A/5B signal pathway, the activated STAT5A/5B dimer in the cytoplasm was required to travel into the nucleus. An eight to ten base pair reverse repetitive DNA sequence known as TTC (C/T) N (G/A) GAA was recognized by the nuclear STAT5A/5B (38). structurally active Mutations of STAT5 caused carcinogenesis *in vitro* and *in vivo* (39). So far, STAT5B mutations are rare and tend only to be found in human myeloid leukemia such as CD4^+^ T-cell prominent granular lymphocytic (T-LGL) leukemia, chronic natural killer lymphoproliferative disorders (CLPD-NK), Acute promyelocytic leukemia (APL) (40, 41). Most mutations in STAT5B occurred in the SH2 region (42). In this study, the mutation frequency of STAT5A in ovarian cancer was found to be extremely low, mainly missense mutation in the SH2 domain **(** **Figure 7** **)**.

In much the same way as other STAT family members, the structural activation of STAT5 contributes to tumor survival, growth, metastasis, and chemotherapy resistance. As mentioned earlier, activated STAT5B is involved in maintaining ovarian CSCs; chemotherapy resistance and tumor immune response are closely related. Nevertheless, little information was available about how STAT5A affected ovarian cancer development and progression. Interestingly, mice carrying STAT5 homozygous deletion (STAT5A−/− 5B −/−) were shown to be sterile, deficient in luteal functional differentiation, and disrupted ovarian development (43). Besides, STAT5 can be activated by various cytokines and hormones. The interaction between steroid receptors such as progesterone and estrogen receptors and nuclear STAT5 stimulates its activity, showing the importance of STAT5 expression in maintaining the ovary’s normal structure and functional integrity (44). According to a study on non-coding RNA transcripts involved in the pathogenesis of ovarian endometriosis (OEM), it was found that STAT5A can be used as a diagnostic marker of OME, and its overexpression was associated with a positive outcome for EOC (45), which was inconsistent with our experimental results. As shown in **Figure 8**, STAT5A expression and ovarian cancer invasion were negatively correlated (R= -0.38). Despite STAT5A/5B being active in most leukemia and some solid tumors, the role of STAT5A/5B in tumor invasion was complicated (46). Data from murine breast cancer studies suggested that STAT5A had dual efficacy in malignant mammary epithelial cells. In the early stage of breast cancer, STAT5A/5B promoted malignant transformation of breast epithelial cells and accelerated tumor growth. In advanced breast cancer, STAT5 was a key molecule regulating and promoting the differentiation of mammary epithelial cells, which can effectively delay the invasion and metastasis of tumors (47). And in the breast cancer clinical sample activated STAT5A/5B was positively correlated with the differentiation status of breast cancer, but it can also prevent the dissemination of confirmed breast cancer, which was a sign of good outcome for breast cancer with negative lymph nodes (48). Phenotypic analysis of TRAM mouse models of prostate cancer and STAT5 knockout mice indicated that STAT5A/5B activation was essential for the growth and survival of prostate cancer. Further studies showed that CyclinD1 and Bcl-xl were the target genes of STAT5 in prostate cancer, which was a potential mechanism of STAT5 regulating prostate cancer (49). Nuclear STAT5A/5B expression predicted early disease recurrence and enhanced the ability of prostate cancer cells to metastasize *in vivo* and *in vitro* (50). Prostate cancer distant clinical metastases were overexpressed with nuclear STAT5A/5B in 61% of cases, which consequently made prostate cancer cells migrate and invade more readily with the aid of microtubule network rearrangement. Importantly, in an experimental *in vivo* metastasis test, activated STAT5 resulted in a ten-fold increase in lung metastasis. In addition, constitutive activation of STAT5 signaling also enhanced cell invasion, migration, and EMT of head and neck squamous cell carcinoma (51). In the subsequent verification, we also confirmed that the expression of STAT5A and P-STAT5A was significantly lower in OV tissues and cell lines, which was closely correlated to the beneficial prognosis in OV patients, especially the low level of P-STAT5A (**Figures 9A–D**). Besides, STAT5A was negatively related to tumor-promoting MMP2 expression in human ovarian serous cystadenocarcinoma cell line HO8910 (**Figures 9D–F**). It is speculated that MMP2 may act as a direct or indirect effect molecule of transcription factor STAT5A to promote the invasion and migration of ovarian cancer, which was in line with previous studies on esophageal cancer (52). Other researchers had suggested that STAT5A activation was related to the regulation of angiogenesis in ovarian cancer, because VEGF secreted by ovarian cancer cells can activate STAT *via* VEGFR in the cancer cells (53). The dual role of activated STAT5A in ovarian cancer invasion demonstrated the complexity of STAT5A function. Of course, we had to admit that there are individual differences among ovarian cancer cell lines, which will be further checked in various human serous cancer cell lines (e.g., SK-OV-3, Shin-3, OVCA-3). We will expand the sample size appropriately to increase the rigor of this validation. In addition, we will construct an ovarian cancer xenograft tumor model and introduce STAT5A or JAK2 recombinant protein to verify the inhibition of MMP2 by high expression of STAT5A, thus affecting the invasion and migration ability of ovarian cancer.

Furthermore, in this study single-cell sequencing data in **Figure 8** also demonstrated that in ovarian cancer cells, STAT5A expression was negatively related to DNA repair (R= -0.36). STAT5A-overexpressed ovarian cancer patients can benefit from multiple types of treatment, including chemotherapy, radiotherapy, and immunotherapy because an essential limiting factor in tumor therapeutic efficacy is tumor cells’ ability to repair DNA damage. In a study of radiation resistance and glutamine anabolism, STAT5 regulated the transcriptional level of glutamine synthetase (GS), then promoted nucleotide metabolism, accelerated DNA damage repair, and eventually made cancer cells more resistant to radiation. In turn, radiation-resistant cells exhibited high glutamine anabolic, including nasopharyngeal carcinoma cells (CNE2-IRR) and glioma cells (U251-IRR) (54). However, a novel class III RKT inhibitor-AIU2001 reduced DNA damage repair genes expression by downregulating STAT5 mRNA level in lung cancer cells (55). Moreover, STAT5A/5B participated in the regulation of DNA repair using homologous recombination in prostate cancer by inducing the RAD51 mRNA level while blocking of JAK2-STAT5A/5B signal pathway sensitized prostate cancer to radiotherapy (56). To sum up, the relationship between STAT5 expression and DNA damage and repair of tumor cells may vary with tumor types.

An integrated prognostic model that includes STAT1, STAT3, STAT4, and STAT5A may be more accurate than one based on a single biomarker. Transcriptional factor families, such as E2F and Forkhead box O (FOXO) transcription factors, have demonstrated outstanding potential as a predictor of cancer outcomes recently. The above studies preliminarily proved that STATs expression had an essential impact on ovarian cancer progression. Mainly, STAT5A affected cell invasion and DNA damage repair, which can be an essential tool to predict ovarian cancer prognosis. However, there are limitations to the current research. Data for this study were derived from the TCGA database and single-cell sequencing, and no independent cohort studies were available. As a next step, we will collect enough clinical samples to validate the effect of STAT family expression on the clinical parameters of ovarian cancer patients.

## Conclusion

Here, a comprehensive analysis of STATs expression and its prognostic value has been carried out to construct an ovarian prognosis model. These results provided a basis for realizing personalized and accurate treatment of ovarian cancer and improving predictive biomarkers. Based on our findings, STAT1, STAT4, and STAT6 may be viable therapeutic targets for ovarian cancer. Low P-STAT5A, but not STAT5A, was a favorable prognostic indicator in human OV. Since STAT5A expression was negatively correlated with ovarian cancer cell invasion and DNA repair, STAT5A/P-STAT5A activators or inducers may increase ovarian cancer survivorship and allow more of them to benefit from radiotherapy and chemotherapy, molecular targeted drug therapy, or immunotherapy.

## Materials and methods

### Source of the data

RNA-sequencing profiles and relevant clinical data consisting of 374 OV tissues came from the TCGA dataset (https://portal.gdc.com). 180 normal control samples were accessed from Genome Type tissue expression (GTEx) datasets (V8) (https://www.gtexportal.org/home/datasets). Additionally, various clinical parameters were collected, including survival status, age, race, pTNM stage, and grade collected in **Table 1**. We used R software v4.0.3 (R Foundation for Statistical Computing, Vienna, Austria) for our statistical analyses. Statistical significance was deemed to be *p*-value <0.05.

The Sankey diagram was constructed with the R software package ggalluvial. The gene mutation data were downloaded and visualized by the map tools package in R software. Genes with higher mutational frequency detected in an ovarian cancer patient in histogram were shown.

### GEPIA2 dataset

A total of 9736 tumors and 8587 normal samples based on the TCGA and GTEx projects were analyzed using the GEPIA2 analyzer. Through the multiple gene comparison columns in the expression analysis plate, the STATs expression level in various tumors was investigated. Besides, we profiled the expression of STATs in the significant stage of ovarian cancer using a box plot in the “pathological stage plot” column.

### Metascape database

Metascape database is a highly effective tool for studying functional gene annotations. Genes and proteins can be analyzed in batches to understand better how genes or proteins work. First, the members of the STAT family were input into the “multiple gene list” text box, and the species “H.sapines” was selected for custom analysis. Findings from the gene ontology enrichment analysis were obtained in the analysis report.

### Kaplan-Meier Plotter

Based on ovarian cancer gene chip data, Kaplan-Meier Plotter analysis was conducted to determine how STAT’s gene expression affects ovarian cancer survival rates. The prognostic value (mainly OS and PFS of ovarian cancer patients) of each member of the STATs family was analyzed, respectively. According to the median values of the expression levels of the samples of the ovarian cancer patients, groups with high and low expression were created. Comparing the two cohorts yielded an HR with 95% confidence intervals (CIs) and a log-rank *P*-value using the Kaplan-Meier survival plot, indicated at the top right of the main graph (57).

### Prognostic value assessment of STATs

The ovarian RNA sequencing data from the TCGA database were converted into transcripts per million (TPM), the data log2 (TPM+1) was normalized, and the clinical information samples were retained for follow-up analysis. An analysis of survival rates among groups by the log-rank test was conducted. The prediction accuracy and risk score of STATs gene were analyzed and compared by time ROC (v0.4).

In this study, the LASSO regression algorithm was employed as a feature selection algorithm, along with 10-fold cross-validation, and a glmnet package in R was performed for the analysis. Multivariate cox regression analysis was used to construct a prognostic model, and first, the multi-factor Cox regression was used to analyze the data, and then the step function performed the iteration. Finally, the optimal model was selected as the final model. Kaplan-Meier curves plotting standard was the same as that described above.

The analyses and R packages were all developed with R (foundation for statistical computing 2020) version 4.0.3. Statistical significance was deemed to be *P*-value <0.05.

### LinkedOmics database

LinkedOmics is a database based on multiple group association data analysis for TCGA. The ovarian cancer data set (TCGA-OV) was selected, and RNAseq was chosen as the data type in the searching and targeting data sets. The target gene STAT5A was input; then, the Pearson Correlation test statistical method was selected for correlation analysis. Finally, we obtained the heat map of the genes positively and negatively related to STAT5A. Moreover, the above results were analyzed by GSEA enrichment analysis in the LinkInterpreter plate based on WebGestalt. In the Enrichment Analysis column, select the KEGG pathway and GO Analysis (Biological process) for further analysis.

### CancerSEA single cell state atlas

The database collects 72 single-cell datasets, totaling 41,900 single cells of 25 human cancers, Mapping the functional states of a single cell of these 14 functional states related to cancer in different cancers. These functional states were also associated with 18,895 protein-coding genes (PCGs) and 15,571 LncRNAs on the single cell level to understand the mechanisms underlying functional differences in cancer cells (58). By inputting the STAT5A gene, the heat map of its correlation with 14 states of ovarian cancer cells was plotted. The status of ovarian cancer cells with a high correlation with the STAT5A gene was filtered by limiting the correlation strength (R > 0.3), and the corresponding scatters plot was generated automatically.

### Cell lines and culture

Human ovarian cancer cell line HO8910 was obtained from the American Type Culture Collection (ATCC). Human normal ovarian epithelial cells (IOSE80) were a kind gift from Hanqing Hong (International Peace Maternity and Child Health Hospital, China). The two kinds of cells were incubated in DMEM High Glucose medium and DMEM-F12medium (HyClone, SH30234.01), respectively, with 10% Foetal Bovine Serum (FBS), 1% penicillin, and 1% streptomycin at 37°C in 5% CO2.

HO8910 cells were transiently transfected with pcDNA3.1-STAT5A-C-3Fla or empty vector plasmids using Lipofectamine™ 3000 Transfection Reagent (Thermo L3000015) according to the manufacturer’s instructions. The total RNA and whole-cell lysates were then harvested for Western blots analysis 48 h after transfection.

### RT-PCR

Total RNA was extracted from HO8910 cells by the Trizol method. According to TAKARA reverse transcription kit instructions, the reaction solution was prepared in a 0.2 mL Ep tube. The reverse transcription conditions were as follows: 37 ℃ for 15 min. The target gene and internal reference gene expression in the cell sample were detected by qPCR. RT^2^Profiler PCR Array Data Analysis system of QIAGEN Company was used for data analysis. Primer sequence for STAT5A and GAPDH (5’to 3’):

STAT5A-human-F: GCAGAGTCCGTGACAGAGG;
STAT5A-human-R: CCACAGGTAGGGACAGAGTCT.
GAPDH-human-F: TCAACGACCACTTTGTCAAGCTCA;
GAPDH-human-R: GCTGGTGGTCCAGGGGTCTTACT.

PCR reaction condition was as follows: 10 min 95°C pre-denaturation; 95°C 15 s, 60°C 60 s PCR cycles for 40 cycles, 60 → 95°C for dissociation curves.

### Western blots

Above 110^7^ IOSE80 and HO8910 cells were collected, respectively. Western blotting analysis was carried out using whole-cell extracts lysed with RIPA lysis buffer (Thermo 89901). The above two cell protein lysates were electrophoresed with 10% sodium dodecyl sulfate-polyacrylamide gel electrophoresis (SDS-PAGE) (Concentration: 100V, 15min; Separation: 120V, 60min) and then transferred to nitrocellulose (NC) membranes (Merck, filter type 0.45μm). Next, all the NC membranes were blocked with 1×protein-free rapid blocking buffer (Epizyme, Shanghai) in a room temperature setting for 40 min. A further incubation step was taken with anti-STAT5A (Proteintech 13179-1-AP), anti-P-STAT5A (Signalway Antibody, 11048), anti-MMP2 (Proteintech, 10373-2-AP), anti-MMP12 (Proteintech, 22989-1-AP), and HRP-conjugated mouse anti-GAPDH (Yeasen Biotech, #30203ES10) diluted to 1:1000 at room temperature for 2 hours. Wash three times in Tris-buffered saline containing 0.1% Tween-20 (TBST) for five minutes each; incubation of the membranes with corresponding secondary antibodies (diluted to 1:10000) followed for 1 hour. The enhanced chemiluminescence (ECL) reagent (Millipore WBKLS0500) was used to visualize protein signals on an Image Quant LAS4000 system (GE Healthcare). These images were analyzed semi-quantitatively using ImageJ 1.8.0 (USA) software, then normalized to a background image.

### Scratch wound assay

HO8910 cells, after different treatments, were seeded in 48-well plates with 1.5x10^5^ cells per well and incubated overnight in DMEM High Glucose medium supplemented with 2%FBS. Then the cell monolayer was scraped horizontally with a 200 μL pipette tip and scratches were immediately generated and washed twice with 1XPBS. 2 ml of fresh DMEM High Glucose medium containing 2% FBS was added, and cells were continued to be cultured for 48 h. Images of cells using an inverted microscope (Leica DMi8) by a 10X objective. Image J software quantified and analyzed the scratch area (freeware http://fiji.sc).

### Transwell invasion assay

The Matrigel was placed in the refrigerator at 4° C overnight from -20° C, and the upper chamber surface of the bottom membrane of the Transwell chamber was coated with 50 mg/L Matrigel (1:8 diluent) and air-dried at 4° C. HO8910 cells after different treatments were digested with trypsin and resuspended with serum-free medium. The cell density was adjusted to 5×10^5^ cells/mL. 200 μL of cell suspension was added into the upper compartment of the Transwell chamber, and 500 μL of culture medium containing 10% FBS was added into the lower chamber of the 24-well plate. The culture plates were placed in a CO_2_ incubator at 37° C for 48 h. The chambers were taken out, and 1 x PBS was washed twice. The cells in the upper layer of the chamber’s membrane were removed carefully using a cotton swab. 4% paraformaldehyde-fixed for 20 min. Crystal violet solution stained for 15 min. Images were taken under an inverted microscope (Leica DMi8) by a 4X objective. 10 fields of view were counted randomly for each sample by Image J software (freeware http://fiji.sc) and then analyzed statistically.

### IHC assay

Ovarian Cancer and adjacent normal tissue microarray (TMA, n=90) were obtained from Shanghai Outdo Biotechnology Company, Ltd (SHXC2021YF01). IHC was carried out as described previously (59). The TMA was placed in an oven at 68°C for 2 h. Dewaxing was completed in the automatic dyeing machine, and the slides were placed in the antigen retrieval instrument to initiate the repair. Then, they were allowed to cool naturally for more than 10 minutes and washed with PBS buffer. The working solution of primary antibodies, including anti-STAT5A (Proteintech 13179-1-AP), anti-P-STAT5A (Signalway Antibody, 11048), anti-MMP2 (Proteintech, 10373-2-AP) diluted 1:200 was added respectively. The slides were kept at 4°C overnight and then rewarmed at room temperature for 45 min, washed with PBS buffer, and put into DAKO automatic IHC instrument. The blocking, secondary antibody binding, and DAB color development procedures were selected according to the “Autostainer Link 48 Use Guide”. The slides were stained with hematoxylin for 1min, immersed in 0.25% alcohol hydrochloric acid (400ml 70% alcohol +1ml concentrated hydrochloric acid) for about 10 s, and rinsed with tap water for 5 min. Then, the slides were dried at room temperature and sealed with neutral resin.

### Statistical analysis

All the experiments were performed independently, at least in triplicate. All the data were expressed as mean ± standard deviation (SD). Statistical analyses were carried out with GraphPad Prism software version 8.0. Multiple group comparisons were performed using a one-way ANOA test. Mann-Whitney test was used to analyze the expression of molecules in tissues. The correlation between molecular and clinical indicators was evaluated by the Chi-square test, Kaplan-Meier survival analysis, and Log-rank Statistical test. *P* < 0.05 was considered statistically significant.

## Data availability statement

The original contributions presented in the study are included in the article/**Supplementary Material**. Further inquiries can be directed to the corresponding authors.

## Author contributions

XG and XL conceived and designed the research. XG performed the experiments, analyzed the data, and wrote the manuscript. Both authors contributed to the article and approved the submitted version.

## Conflict of interest

The authors declare that the research was conducted in the absence of any commercial or financial relationships that could be construed as a potential conflict of interest.

## Publisher’s note

All claims expressed in this article are solely those of the authors and do not necessarily represent those of their affiliated organizations, or those of the publisher, the editors and the reviewers. Any product that may be evaluated in this article, or claim that may be made by its manufacturer, is not guaranteed or endorsed by the publisher.
