# Supplementary material for: In-depth analysis of the expression and functions of signal transducers and activators of transcription in human ovarian cancer
Source: Front Oncol. 2022 Nov 29;12:1054647. doi: 10.3389/fonc.2022.1054647 (PMC9745122; doi:10.3389/fonc.2022.1054647)
Supplement: Supplementary file 1 [file DataSheet_1.docx]

**Supplementary Information**

**In-depth analysis of the expression and functions of signal transducers and activators of transcription in human ovarian cancer**

Xiaodi Gong^1#^, Xiaojun Liu^1#^.

^1^Department of Gynaecology and Obstetrics, Changzheng Hospital, Naval Medical University, 415 Fengyang Road, Shanghai, 200003, China.

^#^ Corresponding author.

E-mail address: [gong­_xdi@sina.com](mailto:gong_xdi@sina.com) (Xiaodi Gong),

[liuxiaojun@smmu.edu.cn. (](mailto:%20%20%20%20%20%20%20%20%20%20%20%20%20liuxiaojun@smmu.edu.cn.%20()Xiaojun Liu).

**Table 1. Top 7 clusters with their representative enriched terms.**

| **GO** | **Description** | **Count** | **%** | **Log10(P)** | **Log10(q)** |
| --- | --- | --- | --- | --- | --- |
| GO:0007259 | receptor signaling pathway via JAK-STAT | 7 | 100 | -20 | -15.77 |
| R-HSA-8854691 | Interleukin-20 family signaling | 6 | 85.71 | -17.93 | -13.94 |
| R-HSA-9020958 | Interleukin-21 signaling | 5 | 71.43 | -16.6 | -12.74 |
| WP2203 | Thymic stromal lymphopoietin (TSLP) signaling pathway | 6 | 85.71 | -16.15 | -12.44 |
| GO:0060397 | growth hormone receptor signaling pathway via JAK-STAT | 4 | 57.14 | -12.67 | -9.55 |
| WP5198 | Inflammatory bowel disease signaling | 4 | 57.14 | -9.91 | -7.19 |
| WP4495 | IL-10 anti-inflammatory signaling pathway | 3 | 42.86 | -8.78 | -6.15 |

"Count" is the number of genes in the STATs lists with membership in the given ontology term. "%" is the percentage of all the STATs lists that are found in the given ontology term. "Log10(P)" is the *P*-value in log base 10. "Log10(q)" is the multi-test adjusted *p*-value in log base 10.

**Table 2. Clinicopathological information of 374 patients with ovarian cancer in TCGA database.**

| **Characteristic** | **Subtype** |  | **No. of cases (%)** |
| --- | --- | --- | --- |
| Status | Alive |  | 144 (38.5) |
|  | Dead |  | 230 (61.5) |
| Age(years) | Mean (SD) | 59.6 (11.4) |  |
|  | Median [MIN, MAX] | 59 [30,87] |  |
|  | <60 |  | 196 (52.4) |
|  | ≥60 |  | 178 (47.5) |
| Race | AMERICAN INDIAN |  | 2 (0.53) |
|  | ASIAN |  | 11 (2.94) |
|  | BLACK |  | 25 (6.68) |
|  | ISLANDER |  | 1 (0.27) |
|  | WHITE |  | 324 (86.6) |
| pTNM_stage | IC |  | 1 (0.27) |
|  | IIA |  | 3 (0.80) |
|  | IIB |  | 3 (0.80) |
|  | IIC |  | 15 (4.01) |
|  | IIIA |  | 7 (1.87) |
|  | IIIB |  | 14 (3.74) |
|  | IIIC |  | 271 (72.4) |
|  | IV |  | 57 (15.2) |
| Grade | G1 |  | 1 (0.27) |
|  | G2 |  | 42 (11.2) |
|  | G3 |  | 320 (85.5) |
|  | G4 |  | 1 (0.27) |
|  | GB |  | 2 (0.53) |
|  | GX |  | 6 (1.60) |

**Table 3. Correlation between the expression of STATs and OS in ovarian cancer patients with different pathological types.**

| **STAT** | **Histological type** | **Case** | **HR (95% CI)** | ***P*-Value** |
| --- | --- | --- | --- | --- |
| STAT1 | All | 1656 | 0.87 (0.76-0.99) | **0.032^*^** |
|  | Serous | 1207 | 0.86 (0.71-0.97) | **0.019^*^** |
|  | Endometrioid | 37 | 1.61 (0.27-9.64) | 0.6 |
| STAT2 | All | 1656 | 0.94 (0.83-1.07) | 0.37 |
|  | Serous | 1207 | 0.96 (0.83-1.12) | 0.62 |
|  | Endometrioid | 37 | 0.53 (0.09-3.18) | 0.29 |
| STAT3 | All | 1656 | 1.04 (0.91-1.18) | 0.59 |
|  | Serous | 1207 | 0.97 (0.83-1.13) | 0.67 |
|  | Endometrioid | 37 | 3.960 (0.44-35.47) | 0.18 |
| STAT4 | All | 1656 | 0.88 (0.77-1) | **0.044^*^** |
|  | Serous | 1207 | 0.89 (0.76-1.03) | 0.12 |
|  | Endometrioid | 37 | 1.11 (0.18-6.62) | 0.91 |
| STAT5A | All | 1656 | 0.93 (0.81-1.05) | 0.24 |
|  | Serous | 1207 | 1.04 (0.89-1.21) | 0.6 |
|  | Endometrioid | 37 | 0.64 (0.11-3.85) | 0.62 |
| STAT5B | All | 1656 | 0.91 (0.8-1.03) | 0.13 |
|  | Serous | 1207 | 1.05 (0.9-1.23) | 0.5 |
|  | Endometrioid | 37 | 0.56 (0.09-3.36) | 0.52 |
| STAT6 | All | 1656 | 0.87 (0.76-0.99) | **0.03^*^** |
|  | Serous | 1207 | 0.87 (0.75-1.02) | 0.083 |
|  | Endometrioid | 37 | 1.23 (0.21-7.37) | 0.82 |

**HR**= hazard ratio, **95% CI**: 95% Confidence interval, **P* <0.05.

**Table 4. Correlation between the expression of STATs and PFS in ovarian cancer patients with different pathological types.**

| **STAT** | **Histological type** | **Case** | **HR (95% CI)** | ***P*-Value** |
| --- | --- | --- | --- | --- |
| STAT1 | All | 1435 | 0.96(0.85-1.09) | 0.57 |
|  | Serous | 1104 | 0.82(0.71-0.95) | **0.0087^**^** |
|  | Endometrioid | 51 | 2.62(0.98-6.99) | **0.046^*^** |
| STAT2 | All | 1435 | 1.06(0.94-1.21) | 0.33 |
|  | Serous | 1104 | 1.29(1.12-1.5) | **0.00046^***^** |
|  | Endometrioid | 51 | 1.41(0.55-3.64) | 0.47 |
| STAT3 | All | 1435 | 1.08（0.95-1.23） | 0.23 |
|  | Serous | 1104 | 0.92(0.8-1.07) | 0.27 |
|  | Endometrioid | 51 | 2.13（0.8-5.71） | 0.12 |
| STAT4 | All | 1435 | 0.99 (0.87-1.12) | 0.83 |
|  | Serous | 1104 | 1.09 (0.94-1.26) | 0.24 |
|  | Endometrioid | 51 | 0.74 (0.29-1.88) | 0.53 |
| STAT5A | All | 1435 | 1.03（0.91-1.17） | 0.62 |
|  | Serous | 1104 | 1.21（1.05-1.4） | **0.0089^**^** |
|  | Endometrioid | 51 | 0.7(0.28-1.78) | 0.45 |
| STAT5B | All | 1435 | 0.97 (0.85-1.1) | 0.6 |
|  | Serous | 1104 | 1.2 (1.04-1.39) | **0.012^*^** |
|  | Endometrioid | 51 | 0.72 (0.28-1.83) | 0.49 |
| STAT6 | All | 1435 | 1.01 (0.89-1.14) | 0.92 |
|  | Serous | 1104 | 1.14 (0.99-1.32) | 0.07 |
|  | Endometrioid | 51 | 2.38 (0.85-6.69) | 0.089 |

**HR**= hazard ratio, **95% CI**: 95% Confidence interval, **P* <0.05, ***P* <0.01, ****P* <0.001.

**Table 5. Correlation between the expression of STATs and PPS in ovarian cancer patients with different pathological types.**

| **STAT** | **Histological type** | **Case** | **HR (95% CI)** | ***P*-Value** |
| --- | --- | --- | --- | --- |
| STAT1 | All | 782 | 0.81(0.68-0.95) | **0.012****^*^** |
|  | Serous | 735 | 0.82(0.69-0.97) | **0.024^*^** |
|  | Endometrioid | 14 | NA | NA |
| STAT2 | All | 782 | 0.98(0.83-1.16) | 0.85 |
|  | Serous | 735 | 1(0.84-1.19) | 0.99 |
|  | Endometrioid | 14 | NA | NA |
| STAT3 | All | 782 | 0.96(0.81-1.13) | 0.6 |
|  | Serous | 735 | 0.93(0.78-1.1) | 0.39 |
|  | Endometrioid | 14 | NA | NA |
| STAT4 | All | 782 | 0.94 (0.79-1.11) | 0.47 |
|  | Serous | 735 | 0.95 (0.8-1.13) | 0.54 |
|  | Endometrioid | 14 | NA | NA |
| STAT5A | All | 782 | 1.04 (0.88-1.23) | 0.62 |
|  | Serous | 735 | 1.08 (0.91-1.28) | 0.39 |
|  | Endometrioid | 14 | NA | NA |
| STAT5B | All | 782 | 1.07 (0.91-1.27) | 0.41 |
|  | Serous | 735 | 1.13 (0.95-1.34) | 0.18 |
|  | Endometrioid | 14 | NA | NA |
| STAT6 | All | 782 | 1.17 (0.99-1.38) | 0.073 |
|  | Serous | 735 | 1.17 (0.98-1.39) | 0.08 |
|  | Endometrioid | 14 | NA | NA |

**HR**= hazard ratio, **95% CI**: 95% Confidence interval, **P* <0.05, NA= not available.

|  |
| --- |
| **Table 6. Variables in the Equation.** |

|  | **B** | **SE** | **Wald** | **df** | **Sig.** | **Exp(B)** | **95% CI for Exp(B)** | |
| --- | --- | --- | --- | --- | --- | --- | --- | --- |
|  |  |  |  |  |  |  | **Lower** | **Upper** |
| TNM_Stage | 3.294 | .862 | 14.61 | 1 | .000 | 26.949 | 4.978 | 145.902 |
| pSTAT5A_Stage | -1.571 | .734 | 4.585 | 1 | .032 | .208 | .049 | .875 |

**B**: Regression coefficient, **SE**：Standard error, **Wald**: Test statistic Wald chi-square value, **df**: Degree of freedom, **Sig.**: Significant (*P* value), **Exp(B)**: Risk ratio (HR value). **95% CI**: 95% Confidence interval.


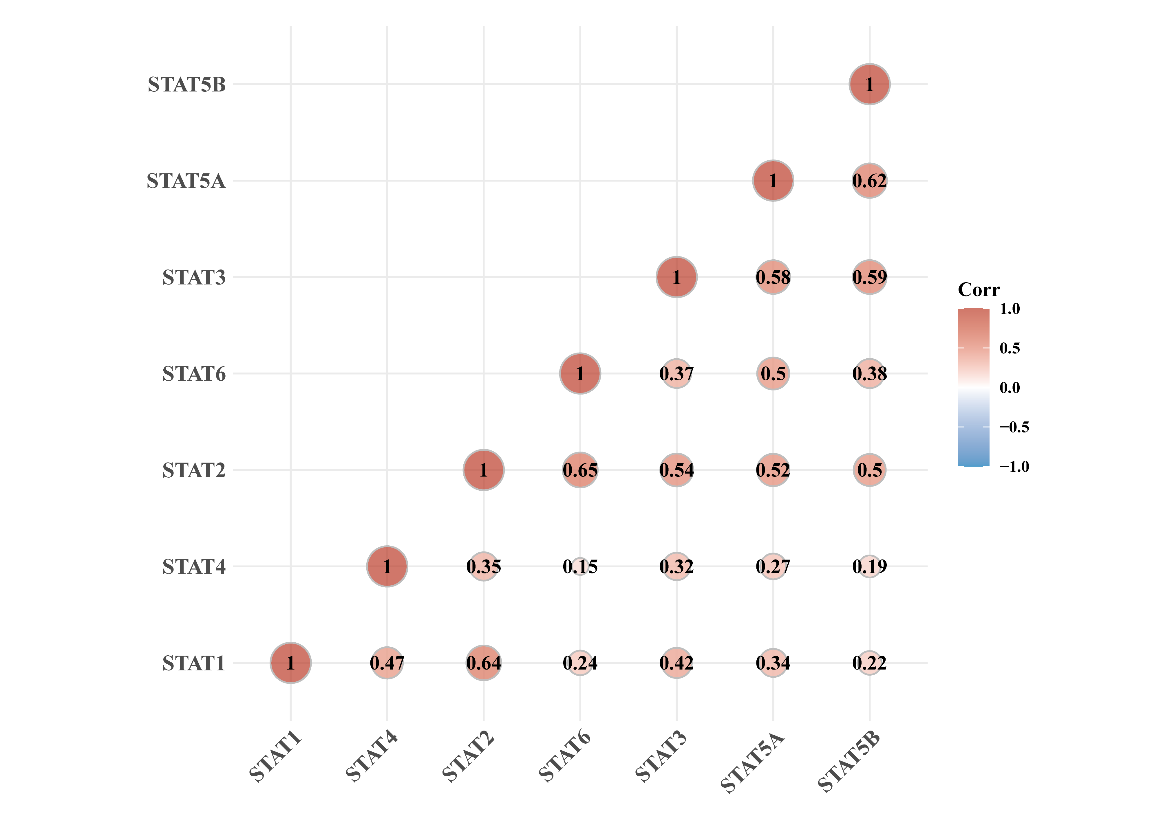


**Figure S1**. **A heatmap of the correlation between members of the STATs family.** Red represents positive correlation whereas blue represents negative correlation. The corresponding Spearman correlation coefficients have been marked in the figure.


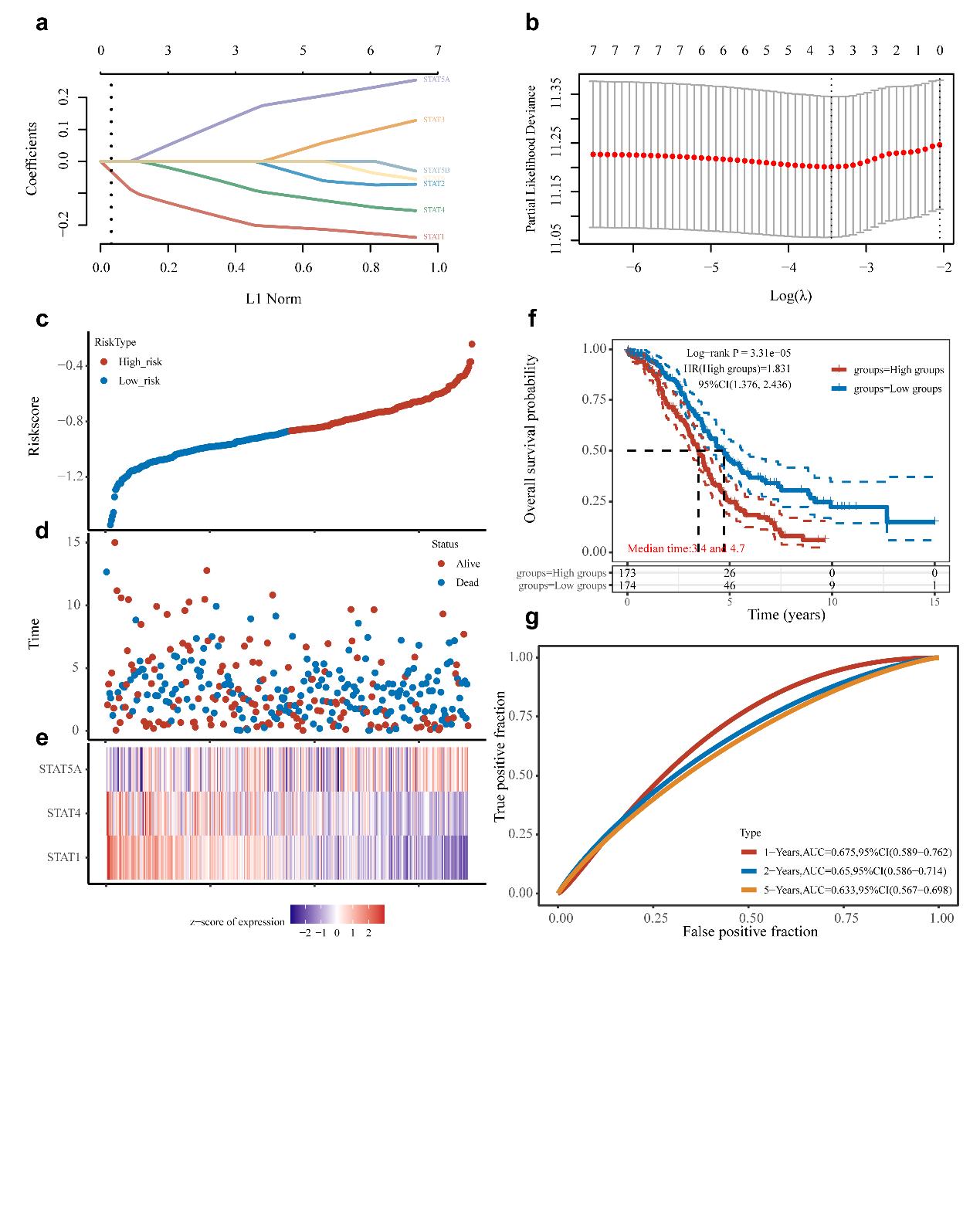


**Figure S2.** **Construction and evaluation of the STAT family prediction model in patients with ovarian cancer.**

a. The coefficients of 7 STATs in LASSO model were screened by a 10-fold cross-validation. b. Analysis of the seven selected STATs by X-tile. c-d. The Risk score, survival time and survival status of selected dataset. e. The heatmap was the gene expression from the signature. f. Kaplan-Meier survival analysis of the risk model of the signature OV patients. HR (High exp) represents the hazard ratio of the low-expression sample relatives to the high-expression sample. g. ROC curves of 1,2,5 years disease-specific survival (DSS) probability based on STATs Risk score.


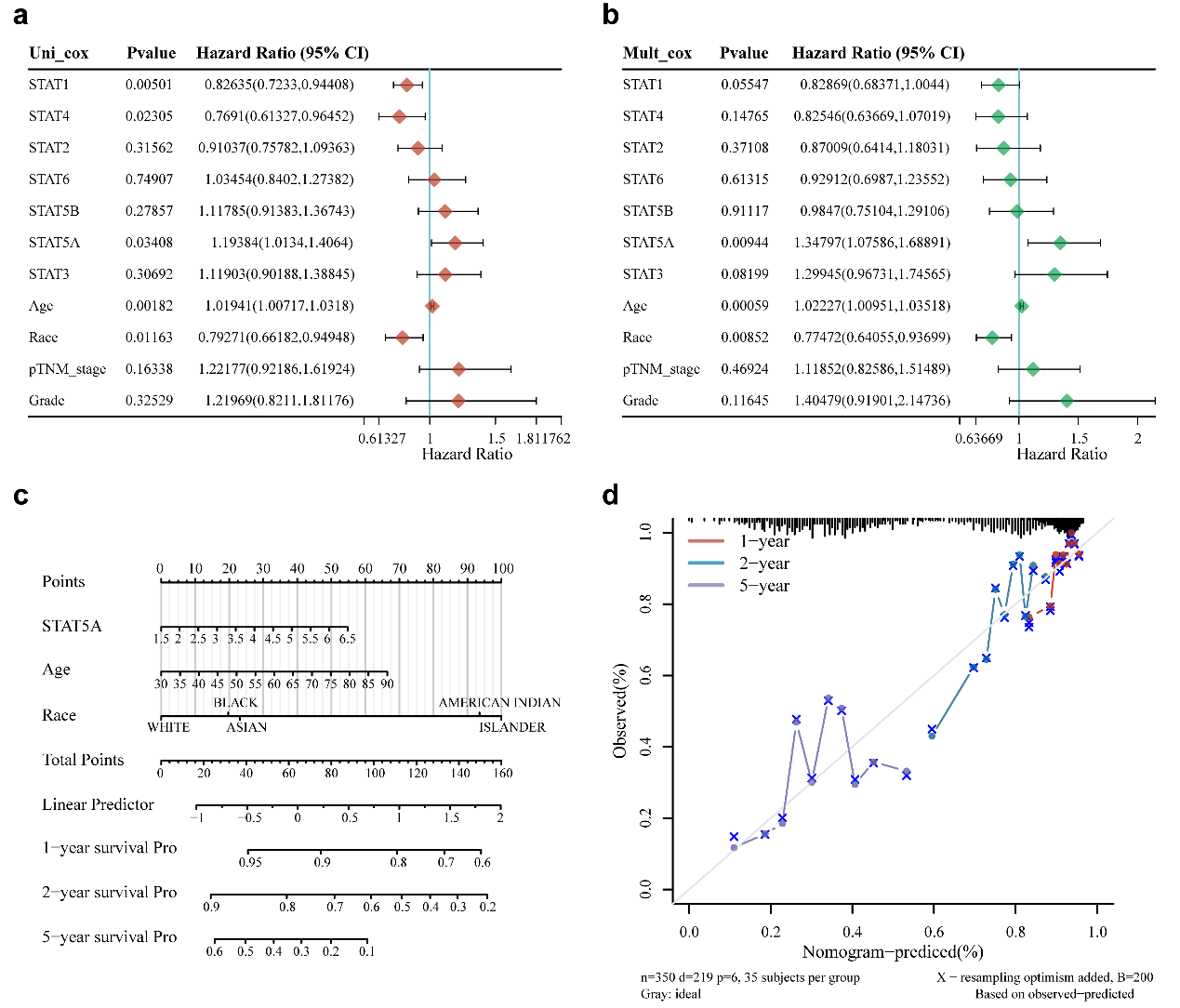


**Figure S3**. **a-b.** Relationship between the expression of STATs genes, clinical characteristics, and OS of patients with ovarian cancer by univariate and multivariate cox regression analysis. **c**. Nomogram for predicting the 1-year, 2-year and 3-year OS of OV cancer patients. d. Calibration curve for the overall survival nomogram model in the discovery group.


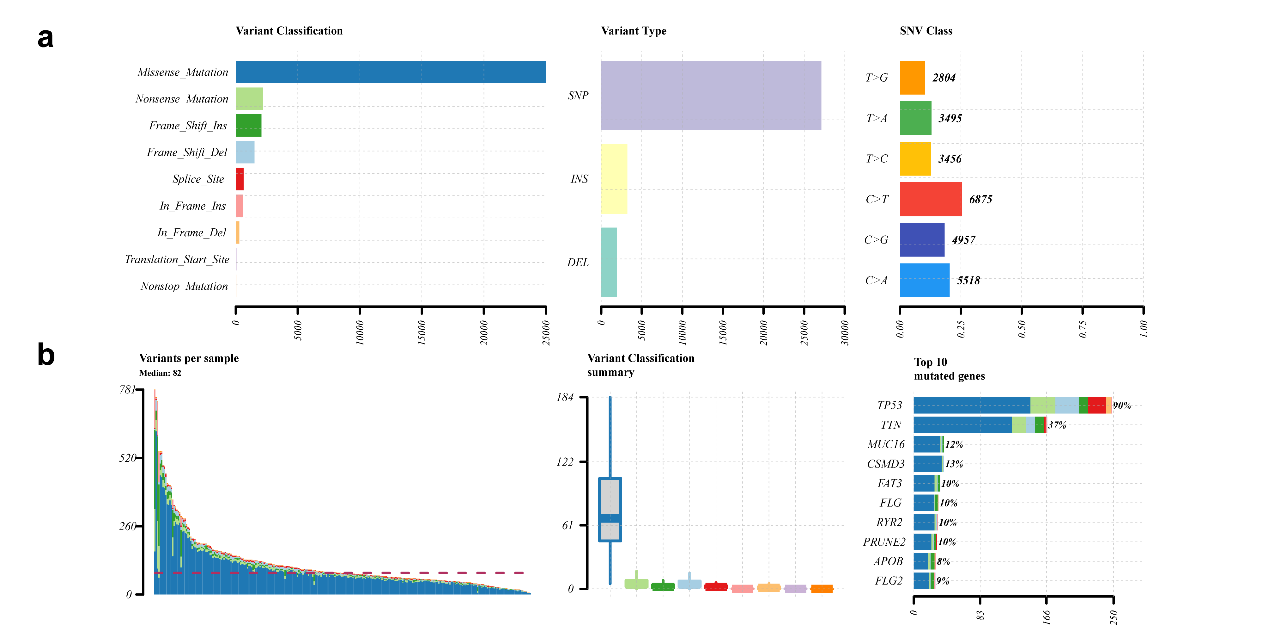


**Figure S4**. **Subgroup analysis of STAT5A gene mutation of OV patients.**

**a.** The distribution of variants in accordance with variant classification, type and single nucleotide variations (SNVs) of OV TCGA cohorts. **b**. The mutation load of each sample (variant classification type), with the stacked bar graph showing the top ten mutated genes.


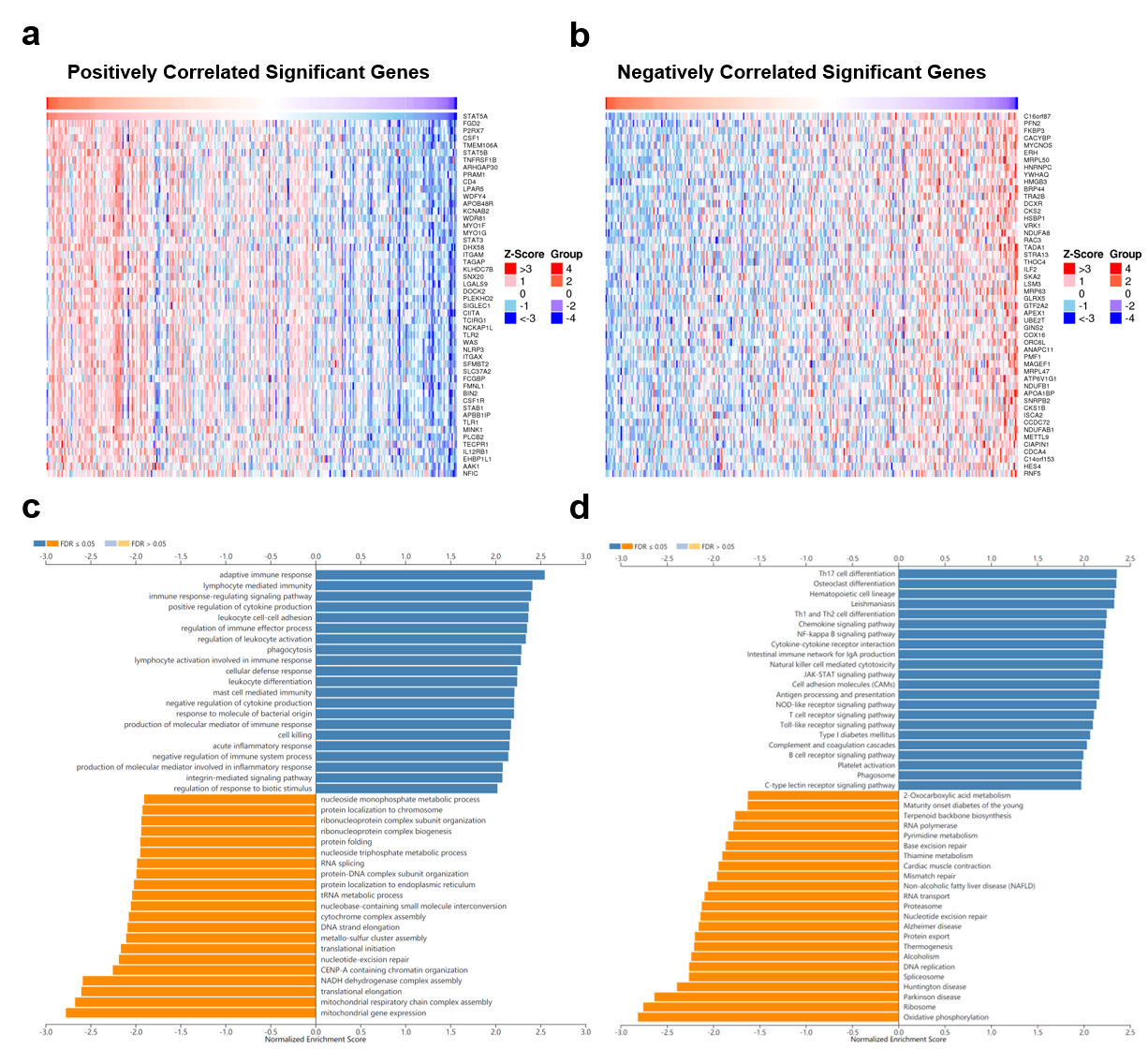


**Figure S5. Enrichment analysis of the function and pathway of STAT5A in OV.**

**a-b.** The heatmap of the differential gene expression associated with STAT5A expression in ovarian cancer. The 50 most positively and negatively affecting genes were both illustrated in this figure. **c-d.** The GO term and KEGG pathway enrichment results for differentially regulated genes.


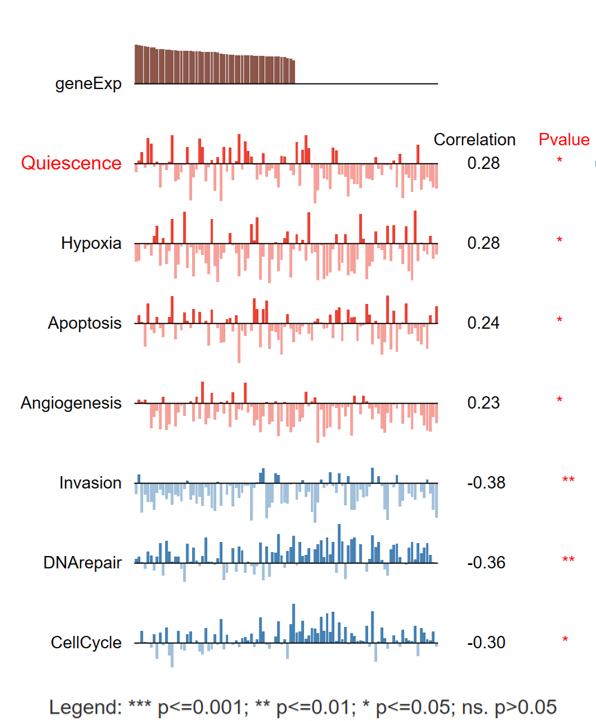


**Figure S6**. **Correlation between STAT5A and 7 functional states of OV cells.**
